# Perspective insights into hydrogels and nanomaterials for ischemic stroke

**DOI:** 10.3389/fncel.2022.1058753

**Published:** 2023-01-24

**Authors:** Qingbo Yu, Zhang Jian, Dan Yang, Tao Zhu

**Affiliations:** ^1^Laboratory of Anesthesia & Critical Care Medicine, Department of Anesthesiology, Translational Neuroscience Center, West China Hospital of Sichuan University, Chengdu, China; ^2^Department of Anesthesiology, North Sichuan Medical College, Nanchong, China; ^3^Sichuan Provincial Maternity and Child Health Care Hospital, Women’s and Children’s Hospital Affiliated of Chengdu Medical College, Chengdu, China

**Keywords:** stroke, biomaterials, hydrogels, nanoparticles, neuroprotection, neurorestoration

## Abstract

Ischemic stroke (IS) is a neurological disorder prevalent worldwide with a high disability and mortality rate. In the clinic setting, tissue plasminogen activator (tPA) and thrombectomy could restore blood flow of the occlusion region and improve the outcomes of IS patients; however, these therapies are restricted by a narrow time window. Although several preclinical trials have revealed the molecular and cellular mechanisms underlying infarct lesions, the translatability of most findings is unsatisfactory, which contributes to the emergence of new biomaterials, such as hydrogels and nanomaterials, for the treatment of IS. Biomaterials function as structural scaffolds or are combined with other compounds to release therapeutic drugs. Biomaterial-mediated drug delivery approaches could optimize the therapeutic effects based on their brain-targeting property, biocompatibility, and functionality. This review summarizes the advances in biomaterials in the last several years, aiming to discuss the therapeutic potential of new biomaterials from the bench to bedside. The promising prospects of new biomaterials indicate the possibility of an organic combination between materialogy and medicine, which is a novel field under exploration.

## Introduction

Stroke is a devastating disease and accounts for 6.7 million deaths per year, according to the World Health Organization (WHO) (Mendis et al., 2015). Ischemic stroke (IS), a fatal cerebrovascular stroke, occurs following sudden brain blood vessel occlusion events. Compared to arterial rupture-induced hemorrhagic stroke and subarachnoid hemorrhage, IS accounted for about 62.4% of all brain strokes in 2019 according to a systematic analysis (GBD Stroke Collaborators, 2021). IS deprives the brain sensorimotor area of nutrients and oxygen, in turn damaging the intracranial parenchyma and contributing to neurological deficits. It also causes a gradual but irreversible neurological damage, leaving most survivors with severe disabilities. In order to minimize brain injury after IS, tissue plasminogen activator (tPA) and surgical thrombectomy are utilized for occluded vessel re-canalization and restoring blood supply (Mendelson and Prabhakaran, 2021). However, due to the narrow treatment time window for tPA (<4.5 h) and thrombectomy (<6 h), only about 10% of IS patients can be effectively treated (Campbell et al., 2019; McMeekin et al., 2021). In addition, for long-term recovery, the limited regenerative capacity of the central nervous system (CNS) is one of the obstacles to the repair of neurological function in stroke patients. Recent advancements in the neuropathological pathways of neurorestoration, neuroprotection, angiogenesis, and brain function recovery reveal several targets for IS, such as neurotrophins, stem cell therapy, and tissue engineering (Burns and Quinones-Hinojosa, 2021; Qin et al., 2022). Hitherto, current strategies have focused on rebuilding reperfusion and protecting the brain from ischemic injury; however, no regenerative medicine is approved for clinical application (Zhang et al., 2020).

In histopathology, IS presents as liquefactive necrosis and damaged brain tissue that eventually progresses as a liquefied cavity without normal tissue structure, making the migration of endogenous reparative cells difficult (Burns and Quinones-Hinojosa, 2021). Moreover, blood-brain barrier (BBB) and blood-cerebrospinal fluid barrier restricts the targeted application of CNS drugs. Since the clinical administration agents for CNS disorders are systemic, the biggest challenge is how to deliver a modest drug at the risk of toxicity. In regenerative medicine, a cell-based therapy for the treatment of IS is known as “the first generation” therapeutic approach. Presently, a series of preliminary clinical trials using neural (NSCs), mesenchymal (MSCs), and hematopoietic stem cells have been conducted to explore their safety, feasibility, and effectiveness (Li M. et al., 2020). Since the current stem cell treatments are administered intravenously, their efficiency depends on the permeability of the BBB. With the progression of IS, the recovery of BBB narrows the time window for cell therapy. Stem cells have been identified as a promising new tool for CNS disorders; however, the safety of the approach lacks evidence and is yet controversial (Boncoraglio et al., 2019).

Emerging tissue engineering focuses on manufacturing medical biomaterials as new interventions to treat stroke lesions (Gallego et al., 2022). Biomaterial-based therapy could transplant therapeutic stem cells or molecules into the targeted brain area rather precisely and with minimal invasion for the treatment of IS (Gallego et al., 2022). In this case, the implantation of biomaterials into the cavity could provide solid structures to attract repair cells or release therapeutic drugs, indicating a possibility for successful clinical translation. A recent meta-analysis reported that intervention using biomaterials, such as scaffolds and particles, contribute to neurological improvement in preclinical stroke models (Bolan et al., 2019). After a brief introduction, the pathological mechanism of IS and the limitations of the current treatment methods and endogenous repair mechanism, the concept of hydrogels and nanomaterials as well as their advantages and disadvantages will be introduced with a focus on the pathophysiological considerations when fabricating the biomaterials for the treatment of IS. Specifically, this review introduces the most recent advance in new biomaterials and their neuroprotection mechanism, providing future perspectives for the effective translation of biomaterials.

## Pathophysiological mechanism of IS

**FIGURE 1:**
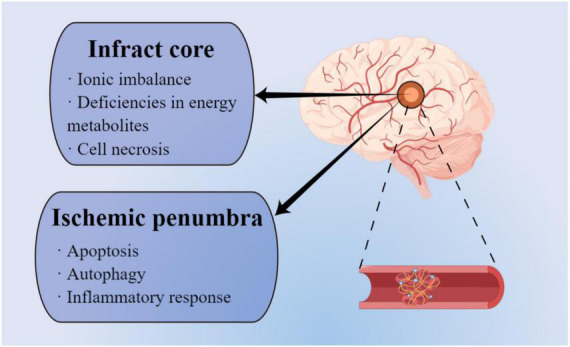
Different pathophysiological mechanisms between infarct area and ischemic penumbra (by figdraw).

When cerebral arteries are occluded by embolism and thrombi, the blood flow supply to the corresponding brain territory is significantly decreased or blocked, leading to the failed energy metabolites delivery and oxygen deprivation, and thus inducing the pathological processes of IS (Kuriakose and Xiao, 2020). The specific neurological impairments such as sensory and functional disorders occur depending on the range of the affected blood flow. On the other hand, based on the severity of the cerebral blood flow reduction, the ischemic lesion area of the brain includes ischemic infarct core and the surrounding ischemic penumbra, both of which have distinct pathophysiological changes (Ermine et al., 2021; Figure 1). Due to the less blood supply in the ischemic core, brain injury is worse than in the ischemic penumbra, which possesses collateral blood vessels circulation. In the ischemic core, ionic imbalance and deficiencies in energy metabolites generate cell necrosis and irreversible cell damage within minutes after an IS event (Tuo et al., 2022). Conversely, ischemic penumbra experience fewer and milder pathological changes, including apoptosis, autophagy, and inflammatory response. Importantly, ischemic penumbra exhibits reversible and dynamic features and is considered a significant clinical target for the treatment of IS in the spotlight (Yang and Liu, 2021).

The multiple complex mechanisms in brain injury after IS involve neuroinflammatory response, oxidative stress (OS) damage, mitochondrial dysfunction, and excitatory neurotoxicity (Qin et al., 2022). In response to acute brain injury, activated M1 phenotype microglia participate in the post-ischemic injury-related immune inflammatory response by producing and secreting inflammatory factors, cell chemokines, and excitatory amino acids (Iadecola et al., 2020). Concurrently, the infiltrated immune cells are recruited from peripheral tissue, resulting in the breakdown of BBB and the inflammatory response cascade (Mastorakos et al., 2021). At the subcellular level, brain hypoxia leads to the depletion of energy substrates required for membrane pumping activity, blocked mitochondrial nicotinamide adenine dinucleotide (NADH) oxidation, and enhanced anaerobic glycolysis (Yang et al., 2018). Subsequently, the increased amount of lactic acid and protons disrupt the acid-base equilibrium, resulting in acidosis and cell membrane rupture. Damaged cells release abundant free radicals and calcium ions, leading to OS damage (Candelario-Jalil et al., 2022). Additionally, excess glutamate and extracellular potassium cause depolarization and hyperexcitability of neurons and glia (Amantea and Bagetta, 2017).

Reperfusion is a primary therapy to reverse brain injury following IS. However, reperfusion itself confers secondary injury to the brain, known as ischemia/reperfusion (I/R) injury. After reperfusion, I/R injuries begin with an oxidative/nitrosative stress peak. Mitochondrial dysfunction is caused by cerebral ischemia injury that hinders the usage of restored oxygen, resulting in the accumulation of oxygen-producing enzymes and reduction of antioxidant enzymes (Jelinek et al., 2021). The imbalance between oxidation and anti-oxidation leads to the accumulation of reactive nitrogen and oxygen species (RNS and ROS, respectively), which magnifies the neuroinflammatory response and induces microvascular disorder termed “no-reflow,” augmenting OS-mediated neuronal death (Chen et al., 2020).

## Current treatment strategies for IS

The standard treatment of IS is the removal of thrombus to relieve cerebral ischemia and hypoxia and restore blood flow or collateral circulation at the earliest to avoid irreversible cell damage or death in the ischemic and peripheral areas (Desai et al., 2021). To date, the only U.S. Food and Drug Administration (FDA)-approved pharmacological therapy for IS treatment is the intravenous administration of tPA (Hollist et al., 2021). Although this protease has demonstrated significant therapeutic effect, the risk of bleeding and the short treatment window (<4.5 h) for tPA intervention makes the majority of patients ineligible for tPA treatment (Saini et al., 2021). Consequently, <5% of IS patients benefit from this only approved drug intervention, according to the statistics (Saini et al., 2021). Endovascular thrombectomy (ET) is another standard therapy for the acute phase of IS. It can be applied alone or in combination with fibrinolytic drugs. Nonetheless, the time window is still limited to 6 h after disease onset (Phipps and Cronin, 2020), and due to the inability of some hospitals to perform ET, only a limited number of patients receive this treatment. Notably, both treatments may lead to disorders of cerebrovascular physiology, which could increase the risk of hemorrhagic transformation (Hong et al., 2021). In addition, patients with successful revascularization also have I/R injury due to high ROS production (Sun et al., 2015). In summary, pre-existing clinical approaches for improving long-term outcomes after IS onset are limited.

Since neurons are terminally differentiated cells, they are difficult to recover and regenerate after ischemic and hypoxic injury. Neuroprotection and neurorestoration have been emphasized repeatedly in the treatment of ischemic brain injury (Chamorro et al., 2021). Previous studies have explored the role of stem cell therapy, anti-inflammatory drugs, antioxidant, and excitotoxicity inhibitors on neuronal plasticity and functional connection (Lyden, 2021). Induced pluripotent stem cells (iPSCs), NSCs, embryonic stem cells (ESCs), and MSCs are common stem cells used in IS treatment in preclinical experiments (Zamorano et al., 2021). Intervening inflammatory chemokine-related signaling pathways, such as chemokine (C-C motif) ligand 2 (CCL2)/CCR2 pathway, could reduce infract volume and monocytes infiltration in the experimental models of IS (Wattananit et al., 2016). Moreover, alleviating OS is a critical therapeutic target, including nuclear factor (erythroid-derived 2)-like 2 (NRF2) and Sirtuin (SIRT)-related signaling pathways (Qin et al., 2022). A recent study reported that *N*-methyl-D-aspartate receptor (NMDAR) antagonist, Memantine, is resistant to glutamate-mediated excitotoxicity and could decrease infract volume without interfering with the normal function of NMDAR (Trotman et al., 2015).

Despite the benefits of these treatments, some limitations involving limited exposure to ischemic brain tissue and short duration of action and retention of therapeutic agents in the brain cannot be ignored. Considering BBB’s natural barrier, novel strategies have been devised to cross this obstacle and deliver therapeutic substances through non-invasive methods (Han and Jiang, 2021). Emerging biomaterials dramatically improve the delivery efficiency of neuroprotective agents to the brain and maintain their progressive release to sustain a constant drug concentration (Parvez et al., 2022).

## Endogenous repair process

The neurovascular unit is composed of vascular cells, neural stem progenitor cells (NSPCs), neurons, glia, and other stem cell subsets and extracellular matrix (ECM) that supports electrical connection, trophic support, structural stability, and interplaying with microvasculature (Wang et al., 2021c). The remodeling of the neurovascular units plays a critical role in the recovery of IS (Ozaki et al., 2019).

Although neurogenesis occurs during embryonic and perinatal development, it has recently been found that NSC proliferation, differentiation, and migration also occur under physiological and pathological conditions during adulthood (Cameron and Glover, 2015; Dillen et al., 2020). In healthy adults, neurogenesis is found in the subgranular zone (SGZ) of the hippocampal and ventricular/subventricular zones (V/SVZ) of the lateral ventricle, from where NSPCs with multipotency could migrate to the olfactory bulb and dentate gyrus to complement olfactory neurons and granular cells, respectively (Zhao and van Praag, 2020). Accumulating evidence from preclinical experiments and post-mortem specimens show enhanced post-stroke neurogenesis (Ceanga et al., 2021). IS significantly increased the production of neuroblasts in the adult brain, and the number of newborn neurons in the iPSCs ilateral striatum was 31 times higher than before. Neurogenesis is activated by attractive factors, growth factors, neurotransmissions, and signal pathways (Chen et al., 2019; Palma-Tortosa et al., 2019). The key role of neuroblasts from SVZ in the post-ischemic neurological function recovery process has been demonstrated *via* transgenic ablation (Jin et al., 2010). Neural stem/progenitor maker-positive cells were detected in the cerebral cortex and ischemic penumbra from the post-stroke autopic human brain (Jin et al., 2006). Despite this phenomenon, the slow endogenous neurogenesis rate and very few surviving high-quality newborn neurons make it difficult to replenish the lesions (Wu et al., 2017). The failure may be due to the adverse microenvironment in lesions, lack of trophic factors, and the deficiency of the broad spectrum of neuronal subtypes (Inta and Gass, 2015). Simultaneously, NPSCs need to migrate from the basal location to the lesion area to survive and proliferate, which might be difficult under the condition of cerebral ischemia and hypoxia (Urban et al., 2019). Other barriers to endogenous repair include gliosis and inflammation, and strengthening endogenous repair strategies could be a path for damaged tissue rebuilding and restoration of neurological function following IS (Xiong et al., 2016).

In addition to neurogenesis, angiogenesis is frequently considered an essential therapeutic target to promote nerve function recovery (Chen et al., 2018; Zong et al., 2020). Normally, blood vessels and axons in the CNS are parallel to each other and have a coupling correlation. Interestingly, neurogenesis and angiogenesis coexist following IS and interact *via* factors, such as vascular endothelial growth factor (VEGF) and basic fibroblast factor (bFGF) secreted by vascular epithelial cells (Ruan et al., 2015). Following neurogenesis in the peri-ischemic core, angiogenesis promotes neuronal survival and contributes to the neuronal plasticity sustained by supplying stable brain perfusion, which in turn contributes to the recovery of nerve function (Yang and Torbey, 2020). During the migration of NSPCs into the ischemic penumbra, angiogenesis generates microvessels that provide oxygen and nutrients and act as scaffolds for neural regeneration by secreting integrins to attract NSPCs (Fujioka et al., 2017, 2019). As interrelated biological processes temporally and spatially, both neurogenesis and angiogenesis participate in the recovery process of injured brain tissue (Rust et al., 2019). However, the ability of spontaneous brain neurogenesis and angiogenesis is limited by few endogenous stem cells and a disturbed microenvironment.

## Biomaterials used for IS treatment

**FIGURE 2:**
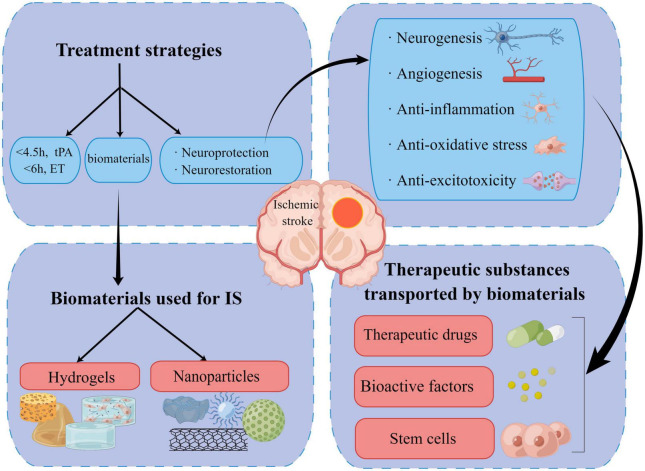
Summary of current therapies, new biomaterials, and the underlying therapeutic mechanism for ischemic stroke (by figdraw).

Since the existing clinical intervention methods and *in vivo* endogenous neurogenesis capability are limited, replacing damaged tissue with exogenous auxiliary stem cells and administering active factors for neuroprotection or stimulating neuronal regeneration based on the original regenerative capacity have become the focus of therapeutics. However, it is difficult to achieve and maintain the ideal dose for sustained neuroprotection with traditional drug delivery methods. Moreover, a few transplanted stem cells fail to survive and re-establish synaptic connections partially due to the disruption of post-ischemic normal physiological environment in the brain. In this scenario, biomaterials have shown outstanding properties in acting as adjuvants and supporting structures for drug/factor and cell delivery (Lv et al., 2022; Figure 2). Biomaterials used for invasive medical therapy include sterilizable nanoparticles (NPs) and hydrogels (Rajkovic et al., 2018). Although distinct mechanical, biological, chemical, and physical features are present, biocompatibility, biofunctionality, and biodegradability are the shared properties for different types of biomaterials (Lin et al., 2022).

### Hydrogels

To improve regenerative efficacy based on limited endogenous repair capacity, natural and synthetic polymer-based hydrogels are engineered as an effective delivery platform owing to their specific physicochemical properties (Tang and Lampe, 2018). Hydrogels are produced from the flexible matrix and insoluble polymers that transform from solution to gel under thermal, physical, and chemical reactions. Therefore, the hydrogels possess a soft and porous three-dimensional (3D) structure with a high-water content that enables their biocompatibility in the soft brain tissue. They act as supporting structures to create an environment for the re-establishment of ECM and migration of new cells and also combine with therapeutic agents or stem cells to suppress inflammation and prevent the formation of the glial scar in peri-lesion or lesion area (Zamorano et al., 2021). A previous study reported that retention of hydrogels derived from ECM for 12 weeks inhibits the progression of stroke cavity and significantly reduces the lesion volume by 28% (Ghuman et al., 2017). Another study reported that hydrogels promote angiogenesis and neurogenesis. The scaffold formed by the interaction between a specific integrin with VEGF-releasing function and hydrogel facilitates non-tortuous vessels regeneration and decreases VEGF-induced vascular permeability (Li et al., 2017). Another study showed that hydrogel-delivered brain-derived neurotrophic factor (BDNF) promotes axonal sprouting in the cortex and corticostriatal system in both mouse and non-human primate stroke models (Cook et al., 2017). Biocompatibility is a typical characteristic of the hydrogel used for scaffolds owing to their high-water content in the composition. Also, their characteristic of biodegradability or bioresorbability protects the patients from long-term local inflammation responses and makes secondary intervention unnecessary.

Natural hydrogels are components of ECM that constitutes one-fifth of the brain parenchyma with the function of maintaining extracellular homeostasis and cell signaling transduction (Samal and Segura, 2021). Natural hydrogels such as alginate, collagen, chitosan, hyaluronic acid (HA), and silk are widely applied for tissue restoration (Table 1). On the other hand, natural hydrogels have advanced anti-inflammatory properties, but the batch-limiting scalability for the brain cavity is challenging. Synthetic hydrogels, such as poly-lactide-co-glycolide (PLGA), polyethylene glycol (PEG), polylactide (PLA), polycaprolactone (PCL), and polyacrylamide (PAM) (Table 1), are biomaterials with advantages of reliable batch production with physiochemical properties, scalability, and chemical modification; however, their use is limited due to low biodegradability (Prestwich et al., 2012). The use of hybrid biomaterials of natural and synthetic hydrogels is rarely reported (Silva et al., 2017).

**TABLE 1:** Summary of different types of hydrogels and nanoparticles.

Hydrogels		Nanoparticles (NPs)
Natural	Synthetic	–
Alginate	Poly-lactide-co-glycolide (PLGA)	Liposomes
Collagen	Polyethylene glycol (PEG)	Polymeric NPs
Chitosan	Polylactide (PLA)	Inorganic NPs
Hyaluronic acid (HA)	Polycaprolactone (PCL)	Other hybrid NPs
Silk	Polyacrylamide (PAM)	–

### NPs

The nanometric scale is 10–500 nm for NPs, which ensures they can interplay with the components inside and outside the cell. Compared to static and steady hydrogels in the injected brain area, NPs present versatile and dynamic features with dispersion and distribution functions in the targeted area. They can be carriers of RNA, DNA, antibodies, bioactive factors, peptides, proteins, and therapeutic compounds (Bharadwaj et al., 2018). In addition, hydrogels combined with NPs are called known as hydrogel NPs which constitute a promising delivery system. Typically, NPs are divided into four types according to the constituent of the core structure: (1) liposomes and other lipid NPs are comprised of fatty acids and triglycerides; (2) polymeric NPs such as natural type (hydrogel NPs: chitosan, gelatin, alginate, and collagen), synthetic type (hydrogel NPs: PLGA, PEG, PLA), nanogels, micelles, and dendrimers; (3) inorganic NPs; and (4) other hybrid NPs (Sarmah et al., 2021; Table 1). Among the NPs above, liposomes and polymers are the most widely used delivery systems.

Liposomes are vesicles formed by an amphiphilic lipid bilayer that has minimal immunogenicity, biocompatibility, and biodegradability due to the analogous constituents with the cell membrane structure (Bharadwaj et al., 2018). Importantly, liposomes are regarded as “the first generation” of drug-delivery nanocarriers, yielding multifaceted therapeutic benefits (Budai and Szogyi, 2001). As liposomes have a water compartment, drugs with hydrophilicity are encapsulated into the core, while hydrophobic substances are embedded within the lipid bilayer. For example, RvD2-loaded neutrophil nanovesicles are constructed using HL-60 cells (human promyelocytic leukemia cells) as vesicle carriers, with lipid-soluble RVD2 bound to the lipid bilayer (Dong et al., 2019). Moreover, the surface modification of the liposome protects the drugs from degrading and clearing out by the immune system before arriving at the target area. Based on the pathological mechanism of IS, the liposome-based biomimetic nanocarriers can be derived from the membranes of platelets, neutrophils, and macrophages (Li M. et al., 2020; Feng et al., 2021; Li et al., 2021).

Compared to liposomes, polymeric NPs have the advantage of stability and tunability. A typical example is PLGA. Owing to its biochemistry properties, PLGA is approved by the US FDA for the treatment of human diseases by being processed into sutures and orthopedic instruments (Ding and Zhu, 2018). A recent finding is that DNA nanostructure-based molecules have the pleiotropic neuroprotection effect (Zhou et al., 2021). Zhou et al. (2021) synthesized tetrahedral framework nucleic acids (tFNAs) from four single-stranded DNAs and demonstrated their ability to cross the BBB after injection through the tail vein.

## Neurorestorative and neuroprotective mechanism of biomaterials

### Angiogenesis

Both VEGF and bFGF are well-known as the most potent proangiogenic factors, and their high expression stimulates the development and maturation of blood vessels (Cecerska-Heryc et al., 2022). Various biomaterials have been used for the angiogenesis and neurological recovery after IS by directly delivering proangiogenic factors or indirectly regulating related upstream pathways and microenvironment (Somaa et al., 2017; Zenych et al., 2021; Yanev et al., 2022; Yin et al., 2022). For example, a histidine-tagged VEGF-laminin-rich sponge is confirmed as a powerful scaffold to enhance the angiogenic activity *in vivo* experiments of mice stroke models (Oshikawa et al., 2017). Another example is the alginate: collagen hydrogel is designed as the carrier vehicle of bFGF to induce angiogenesis (Ali et al., 2018). Also, ECM remodeling is essential during angiogenesis as it provides nourishment and a favorable microenvironment for newborn blood vessels (Ramirez-Calderon et al., 2021). Therefore, well-designed synthetic nanohybrid hydrogels, consisting of sulfated glycosaminoglycan-based polyelectrolyte complex, are developed to provide a native ECM-like bioscaffold for brain tissue regeneration, accelerate NSCs migration, and promote angiogenesis *via* progressive release of bioactive cellular factors, including bFGF and stromal-derived factor-1α (SDF-1α) (Jian et al., 2018). On the other hand, angiogenic factors have adverse effects on their pleiotropic property, in turn leading to increased vascular permeability and worsened brain edema, thus resulting in hemorrhagic transformation (Adamczak and Hoehn, 2015). Due to these challenges, a dual-targeted nanoparticle therapeutic strategy is proposed by combining the ECM integrin ligands and angiogenic factors (Yang H. et al., 2021). In the present study, sonic hedgehog signaling protein smoothed agonist (SAG) coupled to Pro-His-Ser-Arg-Asn (PHSRN) on the hydroxyethyl starch (HES) based nanocarriers platform could be specifically released in the acidic ischemic lesion to favor angiogenesis by activating smoothened (SMO) and transcription factors (Yang H. et al., 2021). In another study, PHSRN, an ECM fibronectin synergistic motif, is proposed as an attractive therapeutic option for the treatment of middle cerebral artery occlusion (MCAO) rats because it triggers angiogenesis by activating VEGF secretion-related upstream pathway and sustains the complexity of survival neurons and induces neurogenesis (Wu et al., 2018). Additionally, the immunomodulatory mechanism plays a pivotal role in hydrogel-based therapy for angiogenesis. When chondroitin sulfate-A (CS-A) hydrogel entrapped with NPCs is implanted into mice stroke models, the protein levels of interleukin-10 (IL-10) and monocyte chemoattractant protein-1 (MCP-1) and the number of PPARγ-positive microglia/macrophages presented a significant increase (McCrary et al., 2022). This phenomenon was further substantiated by an *in vitro* experiment, wherein the microglia/macrophages enwrapped in CS-A produced the proangiogenic and proatherogenic factors (McCrary et al., 2022).

### Neurogenesis and stem cell implantation

Recent studies have shown unprecedented advances in stem cell engineering technology for tissue repair and neurogenesis (Kimbrel and Lanza, 2020). Stimulating the neurogenic potential of endogenous and exogenous stem cell transplantation to constitute the prime therapeutic potential for acute brain injury following IS (Bruggeman et al., 2019). For example, a representative chemotactic signal molecule (SDF-1α aka CXCL12) mediates the migration of MSCs, NSCs, and endothelial progenitor cells (Janssens et al., 2018). The targeted delivery of SDF-1α by pH-sensitive polymer micelle in the infarct area could facilitate neurogenesis and angiogenesis (Kim et al., 2015). Neurotrophic factors may also be pivotal elements of neuronal regeneration and neuroprotection. Hydrogel carried with BDNF, and cerebral dopamine neurotrophic factor (CDNF) exerts therapeutic effects on stroke models of rats (Ravina et al., 2018; Liu X. et al., 2022). In addition to the common factors, erythropoietin (EPO) also plays a fundamental role in neural development and neuroprotection (Wakhloo et al., 2020). *In vivo*, bioengineered local minimally invasive delivery of EPO and epidermal growth factor (EGF) sequentially promotes neurogenesis under IS conditions (Wang et al., 2013). In addition to bioactive factors, an immunosuppressive polypeptide, cyclosporine A (CsA), stimulates the proliferation of brain NSPCs. To avoid the toxicity of CsA systemic application, hyaluronan methylcellulose (HAMC) hydrogels loaded with microspheres containing CsA are injected directly into the cortex of the ischemic area that effectively induce the proliferation of NPSCs in the lateral ventricles (Tuladhar et al., 2015).

Mesenchymal stem cells, iPSCs-derived neural progenitor cells (iPSCs-NPCs), and ESCs-derived NPCs (ESC-NPCs) are the primary seed cells that can be used for stem cell transplantation in the treatment of IS (Stonesifer et al., 2017; Li Z. et al., 2020). Preclinical studies have demonstrated neuronal regenerative and neuroprotective effects using biomaterials pre-seeded with these stem cells (Yan et al., 2015; Somaa et al., 2017; Kim et al., 2020; Wang et al., 2021a). Typically, the optimal delivery conditions and underlying repair mechanisms of the implanted cells are yet under exploration (Wei et al., 2017). Yan et al. (2015) reported that transplantation of MSCs combined with chitosan-collagen scaffold increases the expression of VEGF and nestin-positive NPSCs in the peri-lesion area, DG, and SVZ. To further fit the shape of the lesion cavity, Wang et al. (2021a) improved the tools to transport MSCs based on 3D printing and carbon nanotechnology. Importantly, the production of MSCs, such as extracellular nanovesicles and exosomes, show a promising therapeutic potential for their anti-inflammation and anti-apoptosis ability. In MCAO rats, magnetic nanovesicles from MSCs localized to the infarcted area by magnetic navigation displayed an obvious reduction in infarction size and improved motor function (Kim et al., 2020). Interestingly, a study investigating whether stem cell phenotype affects transplant success showed that less mature stem cells are capable of tissue repair, and mature cells produce unfavorable cell deaths (Payne et al., 2019). Furthermore, stem cell transplantation timing has a significant impact on the outcomes (Doeppner et al., 2014). In summary, Table 2 presents representative bioactive factors and stem cells delivered by biomaterials in the treatment of IS.

**TABLE 2:** Summary of common bioactive factors and stem cells delivered by biomaterials in the treatment of IS.

Bioactive factors/Stem cells	The animal models	Biomaterials	Administration routes	Therapeutic mechanisms	Therapeutic effects	References
BDNF	Permanent dMCAO in rats	Thiol-modified HA hydrogel (HyStem^®^-C)	8 days after surgery, intracranial implantation in the lesion area	Decreasing the number of microglia, astrocytes, and phagocytes in the striatum	Reducing infarct volume and neuroinflammation	Ravina et al., 2018
CDNF	MCAO in rats	Self-assembling peptide RADA16-I hydrogel	7 days after surgery, intracerebroventricular injection	Accelerating the proliferation and migration of NSCs in SVZ and differentiation of NSCs in the ischemic penumbra area of the cerebral cortex by ERK1/2 and STAT3 signal pathway	Neuroprotection, neurogenesis	Liu X. et al., 2022
Ang-1 and VEGF	PTI in cortical area	pH-sensitive peptide nanofiber-based self-assembling hydrogel (PuraMatrix™)	14 days after surgery, lesion center injection	Promoting revascularization and neuronal survival in brain regions surrounding ischemic infarct	Angiogenesis, sensorimotor recovery	Yanev et al., 2022
VEGF	MCAO in rats	CBD-short peptide-PR1P binding VEGF to collagen hydrogels	30 min after MCAO, iPSCs ilateral cerebral cortex injection	Mitigating inflammation response, cell apoptosis, and facilitating angiogenesis and neuronal sparing in the ischemic region of brain	Neuroprotection, angiogenesis	Yin et al., 2022
bFGF and SDF-1α	PTI in rats	Glycosaminoglycan-based hybrid hydrogel encapsulated with polyelectrolyte complex nanoparticles	7 days after surgery, intracranial implantation in lesion area	Recruitment and regulation of endogenous NSCs *via* the signal response of MMP	Enhancing neurogenesis and angiogenesis	Jian et al., 2018
SDF-1α	Permanent MCAO in rats	pH-sensitive polymer micelle PUASM	24 h after surgery, intracranial implantation in iPSCs ilateral striatum	pH-triggered releasing SDF-1α in acid microenvironment of ischemic brain area	Neurogenesis and angiogenesis	Kim et al., 2015
EPO and EGF	ET-1 induced stroke model in mice	HAMC hydrogel loaded with two types of polymeric nanoparticles: EPO in PLGA and pegylated EGF into PLGA core with a poly (sebacic acid) coating	4 days after surgery, intra-epicortical injection	On-demand confining the nanoparticle; in SVZ, increasing the number of NSPCs; in peri-ischemic area, reducing the number of microglia, astrocytes, and macrophagocytes, inhibiting neuronal apoptosis and increasing the number of mature neurons	Neurogenesis and anti-inflammation	Wang et al., 2013
Human ESC-derived cortical progenitor stem cell	ET-1 induced stroke model in rats	SAP hydrogel based on laminin-derived epitope	6 days and 3 weeks after surgery, intracortical injection	Promoting survival of transplanted NPCs and neovascularization; axonal outgrowth and circuit replacement; mitigating cortical atrophy	Progressive sensorimotor improvements	Somaa et al., 2017
Mouse iPSCs-derived NPCs	Sensorimotor cortex mini-stroke model in mice	Chondroitin sulfate-A hydrogel binding bFGF	7 days after surgery, intracranial transplantations in infarct core	Increasing levels of p-STAT3 and p-AKT; and the number of microvessels	Angiogenesis and sensorimotor improvements	McCrary et al., 2020
Human iPSCs derived cortically specified neuroepithelial progenitor cells	ET-1 induced stroke model in rats	HAMC hydrogels	7 days after surgery, intracranial transplantations at cortical lesion regions	Increasing NeuN + host neurons	Neurological function recovery	Payne et al., 2019
Bone marrow MSCs	MCAO in mice	Carbon-nanotubes-doped sericin scaffold based on 3D print technology	2 weeks after surgery, intracranial injection into stroke cavity	Shape-memory graft to fill the cavity; promoting stem cells survival and the differentiation toward mature neurons	Cavity repair	Wang et al., 2021a

Ang1, angiopoietin-1; BDNF, brain-derived neurotrophic factor; bFGF, basic fibroblast factor; CBD, collagen-binding domain; CDNF, cerebral dopamine neurotrophic factor; cNEPs, cortical neuroepithelial progenitor cells; dMCAO, distal middle cerebral artery occlusion; EGF, epidermal growth factor; EPO, erythropoietin; ET-1, endothelin-1; iPSCs, induced pluripotent stem cells; ESC, embryonic stem cells; VEGF, vascular endothelial growth factor; HA, hyaluronic acid; HAMC, hyaluronan methylcellulose; MSCs, mesenchymal stem cells; MMP, matrix metallopeptidase 9; NPCs, neural progenitor cell; NSCs, neural stem cells; NSPCs, neural stem progenitor cell; p-AKT, phosphorylated protein kinase B; PLGA, poly-lactide-co-glycolide; PUASM, polymer poly urethane amino sulfamethazine; PTI, photothrombotic ischemia; SAP, self-assembly of peptides; SDF-1α, stromal-derived factor-1α; STAT, signal transducer and activator of transcription; SVZ, subventricular zone.

### Anti-inflammation

Since long-term inflammatory conditions are the leading detrimental factors to the neurological recovery of post-stroke patients, the anti-inflammatory therapeutics encapsulated in the biomaterials are fabricated to promote neurological repair (Shi et al., 2019). Sword and shield describe the correlation between anti-inflammatory agents and biomaterials. The protective effect of the outer enclosure formed by the biomaterials markedly improves the bioavailability, stability, and druggability of the anti-inflammatory agents (Ross et al., 2015; Ma et al., 2019; Upadhya et al., 2020). Herein, natural compounds, immunosuppressants, and neutrophil-regulating drugs with therapeutic effects on IS stroke transported by NPs are described.

Curcumin is a phytochemical polyphenol composite derived from turmeric root with robust anti-inflammatory and antioxidant properties (Patel et al., 2020). Wang et al. (2019) examined the efficacy of curcumin in treating brain IS injury based on mPEG-b-PLA copolymer NPs. The results revealed that curcumin NPs can easily penetrate the BBB to reach the ischemic areas, hamper the upregulation of M1-type microglia, and reduce the levels of the pro-inflammatory factors, TNF-α and IL-1β, promoting the repair of the damaged BBB (Wang et al., 2019). Immunosuppressants are also the preferred candidates to modulate inflammation after IS (Qiu et al., 2021); for example, mTOR inhibitor rapamycin (RNP) is a potent immunosuppressive agent with anti-inflammatory and anti-proliferative effects (Querfurth and Lee, 2021). Monocytes membrane-coated RNP exerts chemo immunotherapeutic effects with high biosafety by retarding monocyte adhesion to endothelial cells and the recruitment of monocytes (Wang et al., 2021d). In another pathway, decreasing neutrophil infiltration by interfering with the interplay between neutrophil and endothelial cells blocks the inflammatory cascade triggered by the recruitment of peripheral inflammatory cells (Planas, 2018; Jian et al., 2019). Since Resolvin D2 (RvD2) has a strong binding ability with plasma proteins, wrapping RvD2 with neutrophil membrane nanovesicles can drastically improve the transport efficiency to the CNS based on the internal inflammation mechanism that peripheral neutrophils are recruited to the CNS (Dong et al., 2019; Tulowiecka et al., 2020). In the present study, following the binding of RvD2-loaded neutrophil nanovesicles to inflamed brain endothelial cells, inflammation indicating ischemic brain and outcomes of the MCAO mice model improved significantly (Dong et al., 2019).

In addition, targeted neutrophil apoptosis in circulation *via* cytotoxic doxorubicin NPs interrupts neutrophil recruitment and protects post-ischemic brain tissue (Zhang C. Y. et al., 2019). The regulation of the local brain microenvironment aiming at anti-inflammatory has also been regarded as a highlight in the research on brain protection after IS (Liu S. et al., 2022). A previous study showed that polydopamine NPs loaded with monophosphate-adenosine monophosphate synthase (cGAS) inhibitor and CXC chemokine receptor 4 (CXCR4) yield multifaceted benefits to remodel the detrimental microenvironment of the brain in stroke rat (Shi et al., 2022). In the CNS, cGAS inhibitors mediate microglial differentiation into an anti-inflammatory phenotype, and CXCR4 specifically binds to CXCL12 to reduce its chemotactic effect on inflammatory cells, ultimately effectuating the anti-inflammatory reaction, protection, and reduction of infarct size (Shi et al., 2022).

### Anti-oxidative stress

Nanoparticles-based antioxidant includes endogenous anti-oxidases and inorganic nano-reductases, which are widely used to attenuate OS damage by scavenging detrimental free radicals, RNS and ROS (Jiang et al., 2022). Although human-derived endogenous antioxidants exert therapeutic effects by mimicking the natural paradigms, their direct use is hindered by limited cell membrane penetration ability, proteolysis, and poor pharmacokinetics/pharmacodynamics (Song et al., 2021). Hence, nanomaterials with different properties are designed and developed to act as a protective cage for antioxidant substances, facilitating their transport to the site of injury before inactivation and degradation in the *in vivo* conditions (An et al., 2020). For instance, a study exploring the role of nanotechnology-delivered melatonin in the treatment of stroke used a tunneling nanotube to transport melatonin-treated mitochondria into neurons (Yip et al., 2021). Melatonin is a well-known potent endogenous antioxidant, produced by the pineal gland and secreted into circulation (Abolhasanpour et al., 2021). The results showed that melatonin pretreatment preserves mitochondrial integrity and mitochondrial OS by maintaining the stability of mitochondrial electron transport chain complex proteins and increasing the number of mitochondria (Yip et al., 2021). Another common endogenous antioxidant enzyme, superoxide dismutase 1 (SOD1), is designed to be enwrapped by a spherical and hollow nanoparticle based on poly (ethylene glycol)-b-poly (L-lysine)-b-poly (aspartate diethylenetriamine) (PEG-DET) (Jiang et al., 2016). These nanozymes are well-tolerated by neuron and microvascular endothelial cells with low liver and spleen toxicity, and the lesion volume is reduced by half in the mice model of IS (Jiang et al., 2016). Furthermore, the delivery of NPs containing catalase (CAT) and SOD to tPA-treated thromboembolic rat model increases the number of immature neurons or NPSCs by accelerating the OS and inflammation resolution (Petro et al., 2016).

Nonetheless, endogenous antioxidant enzymes suffer from limited substrate types, poor long-term stability, and short circulation half-life (Liang and Yan, 2019). To overcome these problems, various synthetic inorganic antioxidant enzymes, such as ceria oxide, manganese oxide, iron oxide, Prussian blue (PB), and carbogenic nanozymes, have been developed and tested (Poellmann et al., 2018). The first nanozyme to be introduced is cerium oxide (CeO_2_) which has been studied widely. CeO_2_ has a fluorite lattice framework, making it easy to lose oxygen and gain electrons and eliminate hydroxyl radicals and nitric oxide with CAT-like and SOD-like catalytic activities (Naz et al., 2017). Since CeO_2_ possesses an outstanding antioxidant ability, several studies have focused on modifying its surface coating to reduce its biotoxicity and biostability (Saifi et al., 2021). For example, the high biostability and porosity of zeolite doped in cerium can exert dual advantages in a rat model of MCAO (adsorption of zinc ions and resistance to ROS damage), protecting the BBB by hindering the activation of microglia and astrocytes (Huang et al., 2022). Furthermore, the detailed protective mechanism of CeO_2_ NPs on endothelial cells includes attenuating glutamate-induced ROS or decreasing DNA oxidation and mitochondrial superoxide anions (Goujon et al., 2021). However, the catalytic activity of CeO_2_ is mainly dependent on the particle size and the specific surface atomic coordination (Ghorbani et al., 2021). Conversely, manganese dioxide (MnO_2_) and iron oxide (Fe_3_O_4_) are rarely used *in vivo* due to their poor ROS and RNS catalytic efficiency and complex preparation process to be produced into artificial nanozymes (Wu et al., 2020; Yang S. B. et al., 2021). In a recent study, the incorporation of cobalt into Fe_3_O_4_ nanozyme robustly increased the catalytic efficiency of peroxidase and CAT by about 100 times, which is effective in the removal of hydrogen peroxide, superoxide anion, peroxynitrite *in vitro*, and in reducing the lesion size in both focal and permanent stroke models (Liu et al., 2021). To improve the antioxidant effect of MnO_2_, a previous study modulated the OS and inflammatory response by coupling with clinical drugs, such as edaravone or fingolimod, suggesting novel strategies for multi-target combination therapy for stroke (Li et al., 2021; Zhao et al., 2022). Unlike metal oxide with underlying biotoxicity, PB, a nanoparticle with favorable biosafety, has been approved by FDA for detoxification of thallium and cesium poisoning in human body (Busquets and Estelrich, 2020). However, PB cannot cross the BBB *in vivo*. In order to enhance the delivery efficiency, mesoporous PB nanozyme coated with neutrophil-like cell-membrane (MPBzyme@NCM) is developed using biomimetic technology (Feng et al., 2021). In addition to surface coating, the shape and surface area of NPs with enzyme-like activity was schemed carefully to ensure the best therapeutic effects. Hollow PB nanozymes have adequate biosafety and a large specific surface area without obvious side effects (Zhang K. et al., 2019). The *in vivo* and *in vitro* stroke models exerted good antioxidative and anti-inflammatory capability by downregulating the hydroxyl radical generation and transforming ROS and RNS into harmless materials (Zhang K. et al., 2019).

### Anti-excitotoxicity and the other strategies

The overactivation of postsynaptic NMDARs triggers excitotoxicity, leading to excessive accumulation of intracellular calcium and neuronal death (Wu and Tymianski, 2018). Considering that the intrinsic mechanism of NR2B9c to reduce neuroexcitotoxicity is to interfere with the binding of NMDAR to postsynaptic density protein-95 (PSD95), NPs containing NR2B9c peptide are designed to reach the CNS through nasal administration and specifically binding to the cell membrane of neurons in the brain ischemic region of rats using the NPs surface modifier, wheat germ agglutinin (Li et al., 2019). Similarly, drugs that bind to PSD95, such as ZL006, act as protective agents against nerve excitation (Zhao et al., 2016). Under the background of NPs binding T7 peptide and stroke-homing peptide, ZL006 rapidly targets the ischemic area to exert its anti-excitatory effect (Zhao et al., 2016). Also, the positive allosteric regulation of the type A γ-aminobutyric acid (GABA_*A*_) receptors attenuate the neuroexcitatory effect of IS (Liu et al., 2018). Octadecaneuropeptide (ODN), an *in vivo* GABA_*A*_ allosteric molecule secreted by astrocytes, safely and effectively improves the functional recovery after unloading into the stroke core by HA/heparan sulfate proteoglycan hydrogel, as shown in animal experiments (Lamtahri et al., 2021).

Nano photosynthesis therapy is a novel concept in the treatment of IS through the integration of microbial and nanotechnology (Wang et al., 2021b). In this approach, the effective therapeutic components transported by NPs are not the drugs but *S. elongatus*, which acts primarily through oxygen production. Instead of increasing the amount of oxygen carried by hemoglobin in previous studies, the new approach intentionally uses near-infrared light to activate Nd3^+^-doped up converted NPs, turning the light signal into visible light and prompting *S. elongatus* to synthesize oxygen (Wang et al., 2021b). Simultaneously, nanotechnology-mediated gene therapy can be used to enhance the transfection efficiency of therapeutic plasmids for IS treatment. When gene therapy is assisted by self-assembled peptides and heme oxygenase-1 plasmids, they exert cytoprotective effects and reduce infarct volume by inhibiting apoptosis, inflammation, ROS signaling (Oh et al., 2019). In addition to the delivery and scaffold functions mentioned above, nanomaterials can also be used in surgical interventions. For instance, micron-sized nanoknife made from silicon nitride are an emerging concept in neuromicrosurgery. The nanoknife can be used by physicians for precise manipulations at the level of individual axons, generating incisions <100 μ, minimizing and avoiding unnecessary tissue damage (Chang et al., 2007).

## Summary and future perspectives

In a recent study, biomaterial-based formulations for IS recovery have been dedicated to matching the updated pathophysiological mechanism of IS (Liao et al., 2022). In this review, a wide variety of hydrogels and NPs that have been utilized as a treatment strategy for IS are presented and summarized, with emphasis on their superior ability to reconstitute neurovascular units and the characteristics of precise and efficient targeting to the infarct area. Among the new biomaterials in the context, HA hydrogels and liposomes are most widely applied as the desirable bio-scaffolds and nanocarriers with great versatility and outstanding performances (Shahi et al., 2020; Tian et al., 2021). Importantly, biomimetic nanomedicine NPs appear the most promising as practical candidates for clinical translation (Chen et al., 2022).

**FIGURE 3:**
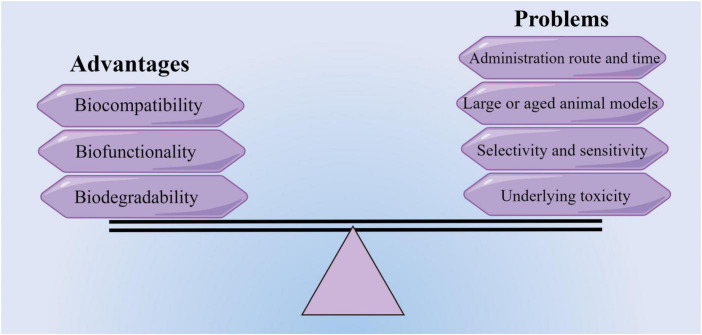
Advantages and potential problems of translating new biomaterials into a potential treatment therapy (by figdraw).

Although the use of biomaterials in characteristic animal models of IS has made some breakthroughs and exhibited beneficial therapeutic effects, the translational trials are in the nascent stage and a long way from clinical application. To date, two different trials testing ECM or collagen hydrogel combined with MSC therapy are currently underway in the early stage to test their safety (NCT04083001 and NCT02767817). Multiple practical issues before the clinical translation of biomaterials must be addressed before being used in the treatment of stroke (Figure 3). Firstly, the size and location of the infarct area may influence the administration route and the formulation of biomaterials. Secondly, stroke has a higher incidence in the elderly and may coexist with other comorbidities in patients, but the models are limited to single diseases based on young-adult rodents. Thirdly, it is necessary to choose the optimal time of administration to achieve the best therapeutic effect (Love et al., 2019; Lyden, 2021). Furthermore, the major stumbling blocks for successful clinical translation also include the lack of long-term behavioral data, the inconsistent recovery measures, and the deficiency in reproducible IS or I/R large animal models, such as non-human primates in preclinical research (Modo et al., 2018; Kaiser and West, 2020).

Drugs incorporated into hydrogels or nanocarriers may arrive at unexpected regions of the brain and have higher dissolution rates beyond control. Briefly, the drawbacks of some biomaterials are the lack of selectivity and sensitivity. Accordingly, an advanced biomaterials delivery system requires further optimization in drug diffusivity, controlled release, targetability to lesion sites, minimizing off-target effects, and biophysical performances, such as the regulation of scaffold mechanics. Thus, future efforts on engineering new biomaterials with remarkable biochemical and biophysical properties for IS treatment should focus on monitoring and avoiding the systematic and neuronal toxicity of biomaterials (Hussain et al., 2020). Taken together, a unified approach or guideline aimed at unequivocally evaluating the toxicity and biosafety of various biomaterials is imperative.

In conclusion, new biomaterials are the most promising replenishments and substitution of limited treatment options available for IS. Moreover, a tailored biomaterial-based therapeutic approach is required for IS under the premise of strict compliance with the regulatory frameworks and obtaining complete pretest data on the safety of the materials.

## Author contributions

QY and ZJ provided and prepared the materials. QY and TZ wrote the manuscript. DY provided the figures. All authors contributed to the article and approved the submitted version.

## References

[B1] Abolhasanpour N. Alihosseini S. Golipourkhalili S. Badalzadeh R. Mahmoudi J. Hosseini L. (2021). Effect of melatonin on endoplasmic reticulum-mitochondrial crosstalk in stroke. *Arch. Med. Res.* 52 673–682. 10.1016/j.arcmed.2021.04.002 33926763

[B2] Adamczak J. Hoehn M. (2015). Poststroke angiogenesis, con: Dark side of angiogenesis. *Stroke* 46 e103–e104. 10.1161/STROKEAHA.114.007642 25813195

[B3] Ali Z. Islam A. Sherrell P. Le-Moine M. Lolas G. Syrigos K. (2018). Adjustable delivery of pro-angiogenic FGF-2 by alginate:collagen microspheres. *Biol. Open* 7:bio027060. 10.1242/bio.027060 29449216 PMC5898261

[B4] Amantea D. Bagetta G. (2017). Excitatory and inhibitory amino acid neurotransmitters in stroke: From neurotoxicity to ischemic tolerance. *Curr. Opin. Pharmacol.* 35 111–119. 10.1016/j.coph.2017.07.014 28826602

[B5] An Z. Yan J. Zhang Y. Pei R. (2020). Applications of nanomaterials for scavenging reactive oxygen species in the treatment of central nervous system diseases. *J. Mater. Chem. B* 8 8748–8767. 10.1039/d0tb01380c 32869050

[B6] Bharadwaj V. N. Nguyen D. T. Kodibagkar V. D. Stabenfeldt S. E. (2018). Nanoparticle-Based therapeutics for brain injury. *Adv. Healthc. Mater.* 7:1700668. 10.1002/adhm.201700668 29034608 PMC5903677

[B7] Bolan F. Louca I. Heal C. Cunningham C. J. (2019). The potential of biomaterial-based approaches as therapies for ischemic stroke: A systematic review and meta-analysis of pre-clinical studies. *Front. Neurol.* 10:924. 10.3389/fneur.2019.00924 31507524 PMC6718570

[B8] Boncoraglio G. B. Ranieri M. Bersano A. Parati E. A. Del G. C. (2019). Stem cell transplantation for ischemic stroke. *Cochrane Database Syst. Rev.* 5:D7231. 10.1002/14651858.CD007231.pub3 31055832 PMC6500737

[B9] Bruggeman K. F. Moriarty N. Dowd E. Nisbet D. R. Parish C. L. (2019). Harnessing stem cells and biomaterials to promote neural repair. *Br. J. Pharmacol.* 176 355–368. 10.1111/bph.14545 30444942 PMC6329623

[B10] Budai M. Szogyi M. (2001). [Liposomes as drug carrier systems. Preparation, classification and therapeutic advantages of liposomes]. *Acta Pharm. Hung.* 71 114–118. 11769091

[B11] Burns T. C. Quinones-Hinojosa A. (2021). Regenerative medicine for neurological diseases-will regenerative neurosurgery deliver? *BMJ* 373:n955. 10.1136/bmj.n955 34162530

[B12] Busquets M. A. Estelrich J. (2020). Prussian blue nanoparticles: Synthesis, surface modification, and biomedical applications. *Drug Discov. Today* 25 1431–1443. 10.1016/j.drudis.2020.05.014 32492486

[B13] Cameron H. A. Glover L. R. (2015). Adult neurogenesis: Beyond learning and memory. *Annu. Rev. Psychol.* 66 53–81. 10.1146/annurev-psych-010814-015006 25251485 PMC5612417

[B14] Campbell B. De Silva D. A. Macleod M. R. Coutts S. B. Schwamm L. H. Davis S. M. (2019). Ischaemic stroke. *Nat. Rev. Dis. Primers* 5:70. 10.1038/s41572-019-0118-8 31601801

[B15] Candelario-Jalil E. Dijkhuizen R. M. Magnus T. (2022). Neuroinflammation, stroke, blood-brain barrier dysfunction, and imaging modalities. *Stroke* 53 1473–1486. 10.1161/STROKEAHA.122.036946 35387495 PMC9038693

[B16] Ceanga M. Dahab M. Witte O. W. Keiner S. (2021). Adult neurogenesis and stroke: A tale of two neurogenic niches. *Front. Neurosci.* 15:700297. 10.3389/fnins.2021.700297 34447293 PMC8382802

[B17] Cecerska-Heryc E. Goszka M. Serwin N. Roszak M. Grygorcewicz B. Heryc R. (2022). Applications of the regenerative capacity of platelets in modern medicine. *Cytokine Growth Factor Rev.* 64 84–94. 10.1016/j.cytogfr.2021.11.003 34924312

[B18] Chamorro A. Lo E. H. Renu A. van Leyen K. Lyden P. D. (2021). The future of neuroprotection in stroke. *J. Neurol. Neurosurg. Psychiatry* 92 129–135. 10.1136/jnnp-2020-324283 33148815

[B19] Chang W. C. Hawkes E. A. Kliot M. Sretavan D. W. (2007). In vivo use of a nanoknife for axon microsurgery. *Neurosurgery* 61 683–691. 10.1227/01.NEU.0000298896.31355.80 17986929

[B20] Chen D. Wei L. Liu Z. R. Yang J. J. Gu X. Wei Z. Z. (2018). Pyruvate kinase m2 increases angiogenesis, neurogenesis, and functional recovery mediated by upregulation of STAT3 and focal adhesion kinase activities after ischemic stroke in adult mice. *Neurotherapeutics* 15 770–784. 10.1007/s13311-018-0635-2 29869055 PMC6095793

[B21] Chen H. He Y. Chen S. Qi S. Shen J. (2020). Therapeutic targets of oxidative/nitrosative stress and neuroinflammation in ischemic stroke: Applications for natural product efficacy with omics and systemic biology. *Pharmacol. Res.* 158:104877. 10.1016/j.phrs.2020.104877 32407958

[B22] Chen J. Jin J. Li K. Shi L. Wen X. Fang F. (2022). Progresses and prospects of neuroprotective agents-loaded nanoparticles and biomimetic material in ischemic stroke. *Front. Cell. Neurosci.* 16:868323. 10.3389/fncel.2022.868323 35480961 PMC9035592

[B23] Chen X. Wu H. Chen H. Wang Q. Xie X. J. Shen J. (2019). Astragaloside VI promotes neural stem cell proliferation and enhances neurological function recovery in transient cerebral ischemic injury via activating EGFR/MAPK signaling cascades. *Mol. Neurobiol.* 56 3053–3067. 10.1007/s12035-018-1294-3 30088176

[B24] Cook D. J. Nguyen C. Chun H. N. Llorente L. I. Chiu A. S. Machnicki M. (2017). Hydrogel-delivered brain-derived neurotrophic factor promotes tissue repair and recovery after stroke. *J. Cereb. Blood Flow Metab.* 37 1030–1045. 10.1177/0271678X16649964 27174996 PMC5363479

[B25] Desai S. M. Jha R. M. Linfante I. (2021). Collateral circulation augmentation and neuroprotection as adjuvant to mechanical thrombectomy in acute ischemic stroke. *Neurology.* 97 (20 Suppl. 2) S178–S184. 10.1212/WNL.0000000000012809 34785616

[B26] Dillen Y. Kemps H. Gervois P. Wolfs E. Bronckaers A. (2020). Adult neurogenesis in the subventricular zone and its regulation after ischemic stroke: Implications for therapeutic approaches. *Transl. Stroke Res.* 11 60–79. 10.1007/s12975-019-00717-8 31309427

[B27] Ding D. Zhu Q. (2018). Recent advances of PLGA micro/nanoparticles for the delivery of biomacromolecular therapeutics. *Mater. Sci. Eng. C Mater. Biol. Appl.* 92 1041–1060. 10.1016/j.msec.2017.12.036 30184728

[B28] Doeppner T. R. Kaltwasser B. Teli M. K. Bretschneider E. Bahr M. Hermann D. M. (2014). Effects of acute versus post-acute systemic delivery of neural progenitor cells on neurological recovery and brain remodeling after focal cerebral ischemia in mice. *Cell Death Dis.* 5:e1386. 10.1038/cddis.2014.359 25144721 PMC4454329

[B29] Dong X. Gao J. Zhang C. Y. Hayworth C. Frank M. Wang Z. (2019). Neutrophil membrane-derived nanovesicles alleviate inflammation to protect mouse brain injury from ischemic stroke. *ACS Nano* 13 1272–1283. 10.1021/acsnano.8b06572 30673266 PMC6424134

[B30] Ermine C. M. Bivard A. Parsons M. W. Baron J. (2021). The ischemic penumbra: From concept to reality. *Int. J. Stroke* 16 497–509. 10.1177/1747493020975229 33818215

[B31] Feng L. Dou C. Xia Y. Li B. Zhao M. Yu P. (2021). Neutrophil-like Cell-membrane-coated nanozyme therapy for ischemic brain damage and long-term neurological functional recovery. *ACS Nano* 15 2263–2280. 10.1021/acsnano.0c07973 33426885

[B32] Fujioka T. Kaneko N. Ajioka I. Nakaguchi K. Omata T. Ohba H. (2017). Beta1 integrin signaling promotes neuronal migration along vascular scaffolds in the post-stroke brain. *EBioMedicine* 16 195–203. 10.1016/j.ebiom.2017.01.005 28153772 PMC5474439

[B33] Fujioka T. Kaneko N. Sawamoto K. (2019). Blood vessels as a scaffold for neuronal migration. *Neurochem. Int.* 126 69–73. 10.1016/j.neuint.2019.03.001 30851365

[B34] Gallego I. Villate-Beitia I. Saenz-Del-Burgo L. Puras G. Pedraz J. L. (2022). Therapeutic opportunities and delivery strategies for brain revascularization in stroke, neurodegeneration, and aging. *Pharmacol. Rev.* 74 439–461. 10.1124/pharmrev.121.000418 35302047

[B35] GBD Stroke Collaborators (2021). Global, regional, and national burden of stroke and its risk factors, 1990-2019: A systematic analysis for the global burden of disease study 2019. *Lancet Neurol.* 20 795–820. 10.1016/S1474-4422(21)00252-0 34487721 PMC8443449

[B36] Ghorbani M. Izadi Z. Jafari S. Casals E. Rezaei F. Aliabadi A. (2021). Preclinical studies conducted on nanozyme antioxidants: Shortcomings and challenges based on US FDA regulations. *Nanomedicine (Lond)* 16 1133–1151. 10.2217/nnm-2021-0030 33973797

[B37] Ghuman H. Gerwig M. Nicholls F. J. Liu J. R. Donnelly J. Badylak S. F. (2017). Long-term retention of ECM hydrogel after implantation into a sub-acute stroke cavity reduces lesion volume. *Acta Biomater.* 63 50–63. 10.1016/j.actbio.2017.09.011 28917705 PMC5653430

[B38] Goujon G. Baldim V. Roques C. Bia N. Seguin J. Palmier B. (2021). Antioxidant activity and toxicity study of cerium oxide nanoparticles stabilized with innovative functional copolymers. *Adv. Healthc. Mater.* 10:e2100059. 10.1002/adhm.202100059 33890419

[B39] Han L. Jiang C. (2021). Evolution of blood-brain barrier in brain diseases and related systemic nanoscale brain-targeting drug delivery strategies. *Acta Pharm. Sin. B* 11 2306–2325. 10.1016/j.apsb.2020.11.023 34522589 PMC8424230

[B40] Hollist M. Morgan L. Cabatbat R. Au K. Kirmani M. F. Kirmani B. F. (2021). Acute stroke management: Overview and recent updates. *Aging Dis.* 12 1000–1009. 10.14336/AD.2021.0311 34221544 PMC8219501

[B41] Hong J. M. Kim D. S. Kim M. (2021). Hemorrhagic transformation after ischemic stroke: Mechanisms and management. *Front. Neurol.* 12:703258. 10.3389/fneur.2021.703258 34917010 PMC8669478

[B42] Huang Z. Qian K. Chen J. Qi Y. E Y. Liang J. (2022). A biomimetic zeolite-based nanoenzyme contributes to neuroprotection in the neurovascular unit after ischaemic stroke via efficient removal of zinc and ROS. *Acta Biomater.* 144 142–156. 10.1016/j.actbio.2022.03.018 35296444

[B43] Hussain Z. Thu H. E. Elsayed I. Abourehab M. Khan S. Sohail M. (2020). Nano-scaled materials may induce severe neurotoxicity upon chronic exposure to brain tissues: A critical appraisal and recent updates on predisposing factors, underlying mechanism, and future prospects. *J. Control. Release* 328 873–894. 10.1016/j.jconrel.2020.10.053 33137366

[B44] Iadecola C. Buckwalter M. S. Anrather J. (2020). Immune responses to stroke: Mechanisms, modulation, and therapeutic potential. *J. Clin. Invest.* 130 2777–2788. 10.1172/JCI135530 32391806 PMC7260029

[B45] Inta D. Gass P. (2015). Is forebrain neurogenesis a potential repair mechanism after stroke? *J Cereb. Blood Flow Metab.* 35 1220–1221. 10.1038/jcbfm.2015.95 25966955 PMC4640286

[B46] Janssens R. Struyf S. Proost P. (2018). The unique structural and functional features of CXCL12. *Cell. Mol. Immunol.* 15 299–311. 10.1038/cmi.2017.107 29082918 PMC6052832

[B47] Jelinek M. Jurajda M. Duris K. (2021). Oxidative stress in the brain: Basic concepts and treatment strategies in stroke. *Antioxidants (Basel)* 10:1886. 10.3390/antiox10121886 34942989 PMC8698986

[B48] Jian W. H. Wang H. C. Kuan C. H. Chen M. H. Wu H. C. Sun J. S. (2018). Glycosaminoglycan-based hybrid hydrogel encapsulated with polyelectrolyte complex nanoparticles for endogenous stem cell regulation in central nervous system regeneration. *Biomaterials* 174 17–30. 10.1016/j.biomaterials.2018.05.009 29763775

[B49] Jian Z. Liu R. Zhu X. Smerin D. Zhong Y. Gu L. (2019). The involvement and therapy target of immune cells after ischemic stroke. *Front. Immunol.* 10:2167. 10.3389/fimmu.2019.02167 31572378 PMC6749156

[B50] Jiang Y. Arounleut P. Rheiner S. Bae Y. Kabanov A. V. Milligan C. (2016). SOD1 nanozyme with reduced toxicity and MPS accumulation. *J. Control. Release* 231 38–49. 10.1016/j.jconrel.2016.02.038 26928528

[B51] Jiang Y. Kang Y. Liu J. Yin S. Huang Z. Shao L. (2022). Nanomaterials alleviating redox stress in neurological diseases: Mechanisms and applications. *J. Nanobiotechnol.* 20:265. 10.1186/s12951-022-01434-5 35672765 PMC9171999

[B52] Jin K. Wang X. Xie L. Mao X. O. Greenberg D. A. (2010). Transgenic ablation of doublecortin-expressing cells suppresses adult neurogenesis and worsens stroke outcome in mice. *Proc. Natl. Acad. Sci. U.S.A.* 107 7993–7998. 10.1073/pnas.1000154107 20385829 PMC2867852

[B53] Jin K. Wang X. Xie L. Mao X. O. Zhu W. Wang Y. (2006). Evidence for stroke-induced neurogenesis in the human brain. *Proc. Natl. Acad. Sci. U.S.A.* 103 13198–13202. 10.1073/pnas.0603512103 16924107 PMC1559776

[B54] Kaiser E. E. West F. D. (2020). Large animal ischemic stroke models: Replicating human stroke pathophysiology. *Neural. Regen. Res.* 15 1377–1387. 10.4103/1673-5374.274324 31997796 PMC7059570

[B55] Kim D. H. Seo Y. K. Thambi T. Moon G. J. Son J. P. Li G. (2015). Enhancing neurogenesis and angiogenesis with target delivery of stromal cell derived factor-1alpha using a dual ionic pH-sensitive copolymer. *Biomaterials* 61 115–125. 10.1016/j.biomaterials.2015.05.025 26001076

[B56] Kim H. Y. Kim T. J. Kang L. Kim Y. J. Kang M. K. Kim J. (2020). Mesenchymal stem cell-derived magnetic extracellular nanovesicles for targeting and treatment of ischemic stroke. *Biomaterials* 243:119942. 10.1016/j.biomaterials.2020.119942 32179302

[B57] Kimbrel E. A. Lanza R. (2020). Next-generation stem cells – ushering in a new era of cell-based therapies. *Nat. Rev. Drug Discov.* 19 463–479. 10.1038/s41573-020-0064-x 32612263

[B58] Kuriakose D. Xiao Z. (2020). Pathophysiology and treatment of stroke: Present status and future perspectives. *Int. J. Mol. Sci.* 21:7609. 10.3390/ijms21207609 33076218 PMC7589849

[B59] Lamtahri R. Hazime M. Gowing E. K. Nagaraja R. Y. Maucotel J. Alasoadura M. (2021). The gliopeptide ODN, a ligand for the benzodiazepine site of GABAA receptors, boosts functional recovery after stroke. *J. Neurosci.* 41 7148–7159. 10.1523/JNEUROSCI.2255-20.2021 34210784 PMC8372017

[B60] Li C. Zhao Z. Luo Y. Ning T. Liu P. Chen Q. (2021). Macrophage-disguised manganese dioxide nanoparticles for neuroprotection by reducing oxidative stress and modulating inflammatory microenvironment in acute ischemic stroke. *Adv. Sci. (Weinh)* 8:e2101526. 10.1002/advs.202101526 34436822 PMC8529435

[B61] Li M. Li J. Chen J. Liu Y. Cheng X. Yang F. (2020). Platelet membrane biomimetic magnetic nanocarriers for targeted delivery and in situ generation of nitric oxide in early ischemic stroke. *ACS Nano* 14 2024–2035. 10.1021/acsnano.9b08587 31927980

[B62] Li Z. Dong X. Tian M. Liu C. Wang K. Li L. (2020). Stem cell-based therapies for ischemic stroke: A systematic review and meta-analysis of clinical trials. *Stem Cell Res. Ther.* 11:252. 10.1186/s13287-020-01762-z 32586371 PMC7318436

[B63] Li R. Huang Y. Chen L. Zhou H. Zhang M. Chang L. (2019). Targeted delivery of intranasally administered nanoparticles-mediated neuroprotective peptide NR2B9c to brain and neuron for treatment of ischemic stroke. *Nanomedicine* 18 380–390. 10.1016/j.nano.2018.10.013 30428334

[B64] Li S. Nih L. R. Bachman H. Fei P. Li Y. Nam E. (2017). Hydrogels with precisely controlled integrin activation dictate vascular patterning and permeability. *Nat. Mater.* 16 953–961. 10.1038/nmat4954 28783156 PMC5809173

[B65] Liang M. Yan X. (2019). Nanozymes: From new concepts, mechanisms, and standards to applications. *Acc. Chem. Res.* 52 2190–2200. 10.1021/acs.accounts.9b00140 31276379

[B66] Liao J. Li Y. Luo Y. Meng S. Zhang C. Xiong L. (2022). Recent advances in targeted nanotherapies for ischemic stroke. *Mol. Pharm.* 19 3026–3041. 10.1021/acs.molpharmaceut.2c00383 35905397

[B67] Lin X. Li N. Tang H. (2022). Recent advances in nanomaterials for diagnosis, treatments, and neurorestoration in ischemic stroke. *Front. Cell. Neurosci.* 16:885190. 10.3389/fncel.2022.885190 35836741 PMC9274459

[B68] Liu J. Zhang J. Wang L. N. (2018). Gamma aminobutyric acid (GABA) receptor agonists for acute stroke. *Cochrane Database Syst. Rev.* 10:D9622. 10.1002/14651858.CD009622.pub5 30376593 PMC6517212

[B69] Liu S. Xu J. Liu Y. You Y. Xie L. Tong S. (2022). Neutrophil-Biomimetic “nanobuffer” for remodeling the microenvironment in the infarct core and protecting neurons in the penumbra via neutralization of detrimental factors to treat ischemic stroke. *ACS Appl. Mater. Interfaces* 14 27743–27761. 10.1021/acsami.2c09020 35695238

[B70] Liu X. Ren H. Peng A. Cheng H. Chen J. Xia X. (2022). The effect of RADA16-I and CDNF on neurogenesis and neuroprotection in brain ischemia-reperfusion injury. *Int. J. Mol. Sci.* 23:1436. 10.3390/ijms23031436 35163360 PMC8836142

[B71] Liu Y. Wang X. Li X. Qiao S. Huang G. Hermann D. M. (2021). A Co-Doped Fe3O4 nanozyme shows enhanced reactive oxygen and nitrogen species scavenging activity and ameliorates the deleterious effects of ischemic stroke. *ACS Appl. Mater. Interfaces* 13 46213–46224. 10.1021/acsami.1c06449 34546708

[B72] Love C. J. Selim M. Spector M. Lo E. H. (2019). Biomaterials for stroke therapy. *Stroke* 50 2278–2284. 10.1161/STROKEAHA.118.023721 31177979

[B73] Lv W. Liu Y. Li S. Lv L. Lu H. Xin H. (2022). Advances of nano drug delivery system for the theranostics of ischemic stroke. *J. Nanobiotechnol.* 20:248. 10.1186/s12951-022-01450-5 35641956 PMC9153106

[B74] Lyden P. D. (2021). Cerebroprotection for acute ischemic stroke: Looking ahead. *Stroke* 52 3033–3044. 10.1161/STROKEAHA.121.032241 34289710 PMC8384682

[B75] Ma Z. Wang N. He H. Tang X. (2019). Pharmaceutical strategies of improving oral systemic bioavailability of curcumin for clinical application. *J. Control. Release* 316 359–380. 10.1016/j.jconrel.2019.10.053 31682912

[B76] Mastorakos P. Mihelson N. Luby M. Burks S. R. Johnson K. Hsia A. W. (2021). Temporally distinct myeloid cell responses mediate damage and repair after cerebrovascular injury. *Nat. Neurosci.* 24 245–258. 10.1038/s41593-020-00773-6 33462481 PMC7854523

[B77] McCrary M. R. Jesson K. Wei Z. Z. Logun M. Lenear C. Tan S. (2020). Cortical transplantation of Brain-mimetic glycosaminoglycan scaffolds and neural progenitor cells promotes vascular regeneration and functional recovery after ischemic stroke in mice. *Adv. Healthc. Mater.* 9:e1900285. 10.1002/adhm.201900285 31977165 PMC7358896

[B78] McCrary M. R. Jiang M. Q. Jesson K. Gu X. Logun M. T. Wu A. (2022). Glycosaminoglycan scaffolding and neural progenitor cell transplantation promotes regenerative immunomodulation in the mouse ischemic brain. *Exp. Neurol.* 357:114177. 10.1016/j.expneurol.2022.114177 35868359 PMC10066865

[B79] McMeekin P. Flynn D. James M. Price C. I. Ford G. A. White P. (2021). Updating estimates of the number of UK stroke patients eligible for endovascular thrombectomy: Incorporating recent evidence to facilitate service planning. *Eur. Stroke J.* 6 349–356. 10.1177/23969873211059471 35342815 PMC8948519

[B80] Mendelson S. J. Prabhakaran S. (2021). Diagnosis and management of transient ischemic attack and acute ischemic stroke: A review. *JAMA* 325 1088–1098. 10.1001/jama.2020.26867 33724327

[B81] Mendis S. Davis S. Norrving B. (2015). Organizational update: The world health organization global status report on noncommunicable diseases 2014; one more landmark step in the combat against stroke and vascular disease. *Stroke* 46 e121–e122. 10.1161/STROKEAHA.115.008097 25873596

[B82] Modo M. M. Jolkkonen J. Zille M. Boltze J. (2018). Future of animal modeling for poststroke tissue repair. *Stroke* 49 1099–1106. 10.1161/STROKEAHA.117.018293 29669872 PMC6013070

[B83] Naz S. Beach J. Heckert B. Tummala T. Pashchenko O. Banerjee T. (2017). Cerium oxide nanoparticles: A ‘radical’ approach to neurodegenerative disease treatment. *Nanomedicine (Lond)* 12 545–553. 10.2217/nnm-2016-0399 28181459

[B84] Oh J. Lee J. Piao C. Jeong J. H. Lee M. (2019). A self-assembled DNA-nanoparticle with a targeting peptide for hypoxia-inducible gene therapy of ischemic stroke. *Biomater. Sci.* 7 2174–2190. 10.1039/c8bm01621f 30900719

[B85] Oshikawa M. Okada K. Kaneko N. Sawamoto K. Ajioka I. (2017). Affinity-Immobilization of VEGF on laminin porous sponge enhances angiogenesis in the ischemic brain. *Adv. Healthc. Mater.* 6:1700183. 10.1002/adhm.201700183 28488337

[B86] Ozaki T. Nakamura H. Kishima H. (2019). Therapeutic strategy against ischemic stroke with the concept of neurovascular unit. *Neurochem. Int.* 126 246–251. 10.1016/j.neuint.2019.03.022 30946849

[B87] Palma-Tortosa S. Hurtado O. Pradillo J. M. Ferreras-Martin R. Garcia-Yebenes I. Garcia-Culebras A. (2019). Toll-like receptor 4 regulates subventricular zone proliferation and neuroblast migration after experimental stroke. *Brain Behav. Immun.* 80 573–582. 10.1016/j.bbi.2019.05.002 31059808

[B88] Parvez S. Kaushik M. Ali M. Alam M. M. Ali J. Tabassum H. (2022). Dodging blood brain barrier with “nano” warriors: Novel strategy against ischemic stroke. *Theranostics* 12 689–719. 10.7150/thno.64806 34976208 PMC8692911

[B89] Patel S. S. Acharya A. Ray R. S. Agrawal R. Raghuwanshi R. Jain P. (2020). Cellular and molecular mechanisms of curcumin in prevention and treatment of disease. *Crit. Rev. Food Sci. Nutr.* 60 887–939. 10.1080/10408398.2018.1552244 30632782

[B90] Payne S. L. Tuladhar A. Obermeyer J. M. Varga B. V. Teal C. J. Morshead C. M. (2019). Initial cell maturity changes following transplantation in a hyaluronan-based hydrogel and impacts therapeutic success in the stroke-injured rodent brain. *Biomaterials* 192 309–322. 10.1016/j.biomaterials.2018.11.020 30468998

[B91] Petro M. Jaffer H. Yang J. Kabu S. Morris V. B. Labhasetwar V. (2016). Tissue plasminogen activator followed by antioxidant-loaded nanoparticle delivery promotes activation/mobilization of progenitor cells in infarcted rat brain. *Biomaterials* 81 169–180. 10.1016/j.biomaterials.2015.12.009 26735970 PMC4715952

[B92] Phipps M. S. Cronin C. A. (2020). Management of acute ischemic stroke. *BMJ* 368:l6983. 10.1136/bmj.l6983 32054610

[B93] Planas A. M. (2018). Role of immune cells migrating to the ischemic brain. *Stroke* 49 2261–2267. 10.1161/STROKEAHA.118.021474 30355002

[B94] Poellmann M. J. Bu J. Hong S. (2018). Would antioxidant-loaded nanoparticles present an effective treatment for ischemic stroke? *Nanomedicine (Lond)* 13 2327–2340. 10.2217/nnm-2018-0084 30284494

[B95] Prestwich G. D. Erickson I. E. Zarembinski T. I. West M. Tew W. P. (2012). The translational imperative: Making cell therapy simple and effective. *Acta Biomater.* 8 4200–4207. 10.1016/j.actbio.2012.06.043 22776825 PMC3488131

[B96] Qin C. Yang S. Chu Y. H. Zhang H. Pang X. W. Chen L. (2022). Signaling pathways involved in ischemic stroke: Molecular mechanisms and therapeutic interventions. *Signal Transduct. Target. Ther.* 7:215. 10.1038/s41392-022-01064-1 35794095 PMC9259607

[B97] Qiu Y. M. Zhang C. L. Chen A. Q. Wang H. L. Zhou Y. F. Li Y. N. (2021). Immune cells in the BBB disruption after acute ischemic stroke: Targets for immune therapy? *Front. Immunol.* 12:678744. 10.3389/fimmu.2021.678744 34248961 PMC8260997

[B98] Querfurth H. Lee H. K. (2021). Mammalian/mechanistic target of rapamycin (mTOR) complexes in neurodegeneration. *Mol. Neurodegener.* 16:44. 10.1186/s13024-021-00428-5 34215308 PMC8252260

[B99] Rajkovic O. Potjewyd G. Pinteaux E. (2018). Regenerative medicine therapies for targeting neuroinflammation after stroke. *Front. Neurol.* 9:734. 10.3389/fneur.2018.00734 30233484 PMC6129611

[B100] Ramirez-Calderon G. Susapto H. H. Hauser C. (2021). Delivery of endothelial cell-laden microgel elicits angiogenesis in self-assembling ultrashort peptide hydrogels in vitro. *ACS Appl. Mater. Interfaces* 13 29281–29292. 10.1021/acsami.1c03787 34142544

[B101] Ravina K. Briggs D. I. Kislal S. Warraich Z. Nguyen T. Lam R. K. (2018). Intracerebral delivery of brain-derived neurotrophic factor using HyStem((R))-C hydrogel implants improves functional recovery and reduces neuroinflammation in a rat model of ischemic stroke. *Int. J. Mol. Sci.* 19:3782. 10.3390/ijms19123782 30486515 PMC6321015

[B102] Ross K. A. Brenza T. M. Binnebose A. M. Phanse Y. Kanthasamy A. G. Gendelman H. E. (2015). Nano-enabled delivery of diverse payloads across complex biological barriers. *J. Control. Release* 219 548–559. 10.1016/j.jconrel.2015.08.039 26315817 PMC4656048

[B103] Ruan L. Wang B. ZhuGe Q. Jin K. (2015). Coupling of neurogenesis and angiogenesis after ischemic stroke. *Brain Res.* 1623 166–173. 10.1016/j.brainres.2015.02.042 25736182 PMC4552615

[B104] Rust R. Gronnert L. Weber R. Z. Mulders G. Schwab M. E. (2019). Refueling the ischemic CNS: Guidance molecules for vascular repair. *Trends Neurosci.* 42 644–656. 10.1016/j.tins.2019.05.006 31285047

[B105] Saifi M. A. Seal S. Godugu C. (2021). Nanoceria, the versatile nanoparticles: Promising biomedical applications. *J. Control. Release* 338 164–189. 10.1016/j.jconrel.2021.08.033 34425166

[B106] Saini V. Guada L. Yavagal D. R. (2021). Global epidemiology of stroke and access to acute ischemic stroke interventions. *Neurology* 97 (20 Suppl. 2) S6–S16. 10.1212/WNL.0000000000012781 34785599

[B107] Samal J. Segura T. (2021). Injectable biomaterial shuttles for cell therapy in stroke. *Brain Res. Bull.* 176 25–42. 10.1016/j.brainresbull.2021.08.002 34391821 PMC8524625

[B108] Sarmah D. Banerjee M. Datta A. Kalia K. Dhar S. Yavagal D. R. (2021). Nanotechnology in the diagnosis and treatment of stroke. *Drug Discov. Today* 26 585–592. 10.1016/j.drudis.2020.11.018 33242696

[B109] Shahi M. Mohammadnejad D. Karimipour M. Rasta S. H. Rahbarghazi R. Abedelahi A. (2020). Hyaluronic acid and regenerative medicine: New insights into the stroke therapy. *Curr. Mol. Med.* 20 675–691. 10.2174/1566524020666200326095837 32213158

[B110] Shi J. Yang Y. Yin N. Liu C. Zhao Y. Cheng H. (2022). Engineering CXCL12 biomimetic decoy-integrated versatile immunosuppressive nanoparticle for ischemic stroke therapy with management of overactivated brain immune microenvironment. *Small Methods* 6:e2101158. 10.1002/smtd.202101158 35041278

[B111] Shi K. Tian D. C. Li Z. G. Ducruet A. F. Lawton M. T. Shi F. D. (2019). Global brain inflammation in stroke. *Lancet Neurol.* 18 1058–1066. 10.1016/S1474-4422(19)30078-X 31296369

[B112] Silva A. D. Aguirre-Cruz L. Guevara J. Ortiz-Islas E. (2017). Nanobiomaterials’ applications in neurodegenerative diseases. *J. Biomater. Appl.* 31 953–984. 10.1177/0885328216659032 28178902

[B113] Somaa F. A. Wang T. Y. Niclis J. C. Bruggeman K. F. Kauhausen J. A. Guo H. (2017). Peptide-Based scaffolds support human cortical progenitor graft integration to reduce atrophy and promote functional repair in a model of stroke. *Cell Rep.* 20 1964–1977. 10.1016/j.celrep.2017.07.069 28834757

[B114] Song G. Zhao M. Chen H. Lenahan C. Zhou X. Ou Y. (2021). The role of nanomaterials in stroke treatment: Targeting oxidative stress. *Oxid. Med. Cell. Longev.* 2021:8857486. 10.1155/2021/8857486 33815664 PMC7990543

[B115] Stonesifer C. Corey S. Ghanekar S. Diamandis Z. Acosta S. A. Borlongan C. V. (2017). Stem cell therapy for abrogating stroke-induced neuroinflammation and relevant secondary cell death mechanisms. *Prog. Neurobiol.* 158 94–131. 10.1016/j.pneurobio.2017.07.004 28743464 PMC5671910

[B116] Sun K. Fan J. Han J. (2015). Ameliorating effects of traditional chinese medicine preparation, chinese materia medica and active compounds on ischemia/reperfusion-induced cerebral microcirculatory disturbances and neuron damage. *Acta Pharm. Sin. B* 5 8–24. 10.1016/j.apsb.2014.11.002 26579420 PMC4629119

[B117] Tang J. D. Lampe K. J. (2018). From de novo peptides to native proteins: Advancements in biomaterial scaffolds for acute ischemic stroke repair. *Biomed. Mater.* 13:34103. 10.1088/1748-605X/aaa4c3 29295967

[B118] Tian X. Fan T. Zhao W. Abbas G. Han B. Zhang K. (2021). Recent advances in the development of nanomedicines for the treatment of ischemic stroke. *Bioact. Mater.* 6 2854–2869. 10.1016/j.bioactmat.2021.01.023 33718667 PMC7905263

[B119] Trotman M. Vermehren P. Gibson C. L. Fern R. (2015). The dichotomy of memantine treatment for ischemic stroke: Dose-dependent protective and detrimental effects. *J. Cereb. Blood Flow Metab.* 35 230–239. 10.1038/jcbfm.2014.188 25407270 PMC4426739

[B120] Tuladhar A. Morshead C. M. Shoichet M. S. (2015). Circumventing the blood-brain barrier: Local delivery of cyclosporin a stimulates stem cells in stroke-injured rat brain. *J. Control. Release* 215 1–11. 10.1016/j.jconrel.2015.07.023 26226344

[B121] Tulowiecka N. Kotlega D. Prowans P. Szczuko M. (2020). The role of resolvins: EPA and DHA derivatives can be useful in the prevention and treatment of ischemic stroke. *Int. J. Mol. Sci.* 21:7628. 10.3390/ijms21207628 33076354 PMC7589657

[B122] Tuo Q. Z. Zhang S. T. Lei P. (2022). Mechanisms of neuronal cell death in ischemic stroke and their therapeutic implications. *Med. Res. Rev.* 42 259–305. 10.1002/med.21817 33957000

[B123] Upadhya R. Zingg W. Shetty S. Shetty A. K. (2020). Astrocyte-derived extracellular vesicles: Neuroreparative properties and role in the pathogenesis of neurodegenerative disorders. *J. Control. Release* 323 225–239. 10.1016/j.jconrel.2020.04.017 32289328 PMC7299747

[B124] Urban N. Blomfield I. M. Guillemot F. (2019). Quiescence of adult mammalian neural stem cells: A highly regulated rest. *Neuron* 104 834–848. 10.1016/j.neuron.2019.09.026 31805262

[B125] Wakhloo D. Scharkowski F. Curto Y. Javed B. U. Bansal V. Steixner-Kumar A. A. (2020). Functional hypoxia drives neuroplasticity and neurogenesis via brain erythropoietin. *Nat. Commun.* 11:1313. 10.1038/s41467-020-15041-1 32152318 PMC7062779

[B126] Wang L. Xiong X. Zhang L. Shen J. (2021c). Neurovascular unit: A critical role in ischemic stroke. *CNS Neurosci. Ther.* 27 7–16. 10.1111/cns.13561 33389780 PMC7804897

[B127] Wang J. Li X. Song Y. Su Q. Xiaohalati X. Yang W. (2021a). Injectable silk sericin scaffolds with programmable shape-memory property and neuro-differentiation-promoting activity for individualized brain repair of severe ischemic stroke. *Bioact. Mater.* 6 1988–1999. 10.1016/j.bioactmat.2020.12.017 33474513 PMC7786039

[B128] Wang Y. Wang Y. Li S. Cui Y. Liang X. Shan J. (2021d). Functionalized nanoparticles with monocyte membranes and rapamycin achieve synergistic chemoimmunotherapy for reperfusion-induced injury in ischemic stroke. *J. Nanobiotechnol.* 19:331. 10.1186/s12951-021-01067-0 34674712 PMC8529766

[B129] Wang J. Su Q. Lv Q. Cai B. Xiaohalati X. Wang G. (2021b). Oxygen-generating cyanobacteria powered by upconversion-nanoparticles-converted near-infrared light for ischemic stroke treatment. *Nano Lett.* 21 4654–4665. 10.1021/acs.nanolett.1c00719 34008994

[B130] Wang Y. Cooke M. J. Sachewsky N. Morshead C. M. Shoichet M. S. (2013). Bioengineered sequential growth factor delivery stimulates brain tissue regeneration after stroke. *J. Control. Release* 172 1–11. 10.1016/j.jconrel.2013.07.032 23933523

[B131] Wang Y. Luo J. Li S. Y. (2019). Nano-curcumin simultaneously protects the blood-brain barrier and reduces m1 microglial activation during cerebral Ischemia-Reperfusion injury. *ACS Appl. Mater. Interfaces* 11 3763–3770. 10.1021/acsami.8b20594 30618231

[B132] Wattananit S. Tornero D. Graubardt N. Memanishvili T. Monni E. Tatarishvili J. (2016). Monocyte-derived macrophages contribute to spontaneous long-term functional recovery after stroke in mice. *J. Neurosci.* 36 4182–4195. 10.1523/JNEUROSCI.4317-15.2016 27076418 PMC6601783

[B133] Wei L. Wei Z. Z. Jiang M. Q. Mohamad O. Yu S. P. (2017). Stem cell transplantation therapy for multifaceted therapeutic benefits after stroke. *Prog. Neurobiol.* 157 49–78. 10.1016/j.pneurobio.2017.03.003 28322920 PMC5603356

[B134] Wu C. C. Wang L. C. Su Y. T. Wei W. Y. Tsai K. J. (2018). Synthetic alpha5beta1 integrin ligand PHSRN is proangiogenic and neuroprotective in cerebral ischemic stroke. *Biomaterials* 185 142–154. 10.1016/j.biomaterials.2018.09.014 30243150

[B135] Wu K. J. Yu S. Lee J. Y. Hoffer B. Wang Y. (2017). Improving neurorepair in stroke brain through endogenous neurogenesis-enhancing drugs. *Cell Transplant.* 26 1596–1600. 10.1177/0963689717721230 29113469 PMC5680955

[B136] Wu M. R. Lee C. H. Hsiao J. K. (2020). Bidirectional enhancement of cell proliferation between iron oxide nanoparticle-labeled mesenchymal stem cells and choroid plexus in a cell-based therapy model of ischemic stroke. *Int J Nanomedicine.* 15 9181–9195. 10.2147/IJN.S278687 33239875 PMC7682617

[B137] Wu Q. J. Tymianski M. (2018). Targeting NMDA receptors in stroke: New hope in neuroprotection. *Mol. Brain* 11:15. 10.1186/s13041-018-0357-8 29534733 PMC5851248

[B138] Xiong X. Y. Liu L. Yang Q. W. (2016). Functions and mechanisms of microglia/macrophages in neuroinflammation and neurogenesis after stroke. *Prog. Neurobiol.* 142 23–44. 10.1016/j.pneurobio.2016.05.001 27166859

[B139] Yan F. Yue W. Zhang Y. L. Mao G. C. Gao K. Zuo Z. X. (2015). Chitosan-collagen porous scaffold and bone marrow mesenchymal stem cell transplantation for ischemic stroke. *Neural. Regen. Res.* 10 1421–1426. 10.4103/1673-5374.163466 26604902 PMC4625507

[B140] Yanev P. van Tilborg G. A. van der Toorn A. Kong X. Stowe A. M. Dijkhuizen R. M. (2022). Prolonged release of VEGF and Ang1 from intralesionally implanted hydrogel promotes perilesional vascularization and functional recovery after experimental ischemic stroke. *J. Cereb. Blood Flow Metab.* 42 1033–1048. 10.1177/0271678X211069927 34986707 PMC9125493

[B141] Yang H. Luo Y. Hu H. Yang S. Li Y. Jin H. (2021). PH-Sensitive, cerebral vasculature-targeting hydroxyethyl starch functionalized nanoparticles for improved angiogenesis and neurological function recovery in ischemic stroke. *Adv. Healthc. Mater.* 10:e2100028. 10.1002/adhm.202100028 34028998

[B142] Yang S. B. Li X. L. Li K. Zhang X. X. Yuan M. Guo Y. S. (2021). The colossal role of H-MnO2-PEG in ischemic stroke. *Nanomedicine* 33:102362. 10.1016/j.nano.2021.102362 33476765

[B143] Yang J. L. Mukda S. Chen S. D. (2018). Diverse roles of mitochondria in ischemic stroke. *Redox Biol.* 16 263–275. 10.1016/j.redox.2018.03.002 29549824 PMC5854930

[B144] Yang S. H. Liu R. (2021). Four decades of ischemic penumbra and its implication for ischemic stroke. *Transl. Stroke Res.* 12 937–945. 10.1007/s12975-021-00916-2 34224106 PMC8557134

[B145] Yang Y. Torbey M. T. (2020). Angiogenesis and blood-brain barrier permeability in vascular remodeling after stroke. *Curr. Neuropharmacol.* 18 1250–1265. 10.2174/1570159X18666200720173316 32691713 PMC7770645

[B146] Yin J. Shi C. He W. Yan W. Deng J. Zhang B. (2022). Specific bio-functional CBD-PR1P peptide binding VEGF to collagen hydrogels promotes the recovery of cerebral ischemia in rats. *J. Biomed. Mater. Res. A* 110 1579–1589. 10.1002/jbm.a.37409 35603700

[B147] Yip H. K. Dubey N. K. Lin K. C. Sung P. H. Chiang J. Y. Chu Y. C. (2021). Melatonin rescues cerebral ischemic events through upregulated tunneling nanotube-mediated mitochondrial transfer and downregulated mitochondrial oxidative stress in rat brain. *Biomed. Pharmacother.* 139:111593. 10.1016/j.biopha.2021.111593 33865018

[B148] Zamorano M. Castillo R. L. Beltran J. F. Herrera L. Farias J. A. Antileo C. (2021). Tackling ischemic reperfusion injury with the aid of stem cells and tissue engineering. *Front Physiol.* 12:705256. 10.3389/fphys.2021.705256 34603075 PMC8484708

[B149] Zenych A. Jacqmarcq C. Aid R. Fournier L. Forero R. L. Chaubet F. (2021). Fucoidan-functionalized polysaccharide submicroparticles loaded with alteplase for efficient targeted thrombolytic therapy. *Biomaterials* 277:121102. 10.1016/j.biomaterials.2021.121102 34482087

[B150] Zhang C. Y. Dong X. Gao J. Lin W. Liu Z. Wang Z. (2019). Nanoparticle-induced neutrophil apoptosis increases survival in sepsis and alleviates neurological damage in stroke. *Sci Adv.* 5:x7964. 10.1126/sciadv.aax7964 31723603 PMC6834394

[B151] Zhang K. Tu M. Gao W. Cai X. Song F. Chen Z. (2019). Hollow prussian blue nanozymes drive neuroprotection against ischemic stroke via attenuating oxidative stress, counteracting inflammation, and suppressing cell apoptosis. *Nano Lett.* 19 2812–2823. 10.1021/acs.nanolett.8b04729 30908916

[B152] Zhang S. Lachance B. B. Moiz B. Jia X. (2020). Optimizing stem cell therapy after ischemic brain Injury. *J. Stroke* 22 286–305. 10.5853/jos.2019.03048 33053945 PMC7568970

[B153] Zhao Q. Du W. Zhou L. Wu J. Zhang X. Wei X. (2022). Transferrin-enabled blood-brain barrier crossing manganese-based nanozyme for rebalancing the reactive oxygen species level in ischemic stroke. *Pharmaceutics* 14:1122. 10.3390/pharmaceutics14061122 35745695 PMC9231148

[B154] Zhao X. van Praag H. (2020). Steps towards standardized quantification of adult neurogenesis. *Nat. Commun.* 11:4275. 10.1038/s41467-020-18046-y 32848155 PMC7450090

[B155] Zhao Y. Jiang Y. Lv W. Wang Z. Lv L. Wang B. (2016). Dual targeted nanocarrier for brain ischemic stroke treatment. *J. Control. Release* 233 64–71. 10.1016/j.jconrel.2016.04.038 27142584

[B156] Zhou M. Zhang T. Zhang B. Zhang X. Gao S. Zhang T. (2021). A DNA Nanostructure-based neuroprotectant against neuronal Apoptosis via inhibiting toll-like receptor 2 signaling pathway in acute ischemic stroke. *ACS Nano* 16 1456–1470. 10.1021/acsnano.1c09626 34967217

[B157] Zong X. Li Y. Liu C. Qi W. Han D. Tucker L. (2020). Theta-burst transcranial magnetic stimulation promotes stroke recovery by vascular protection and neovascularization. *Theranostics* 10 12090–12110. 10.7150/thno.51573 33204331 PMC7667689

